# Salmonellosis in the Republic of Georgia: using molecular typing to identify the outbreak-causing strain.

**DOI:** 10.3201/eid0601.000113

**Published:** 2000

**Authors:** A Sulakvelidze, M Kekelidze, D Turabelidze, S Tsanava, L Tevsadze, L Devdariani, R Gautom, R Myers, J G Morris, P Imnadze

**Affiliations:** University of Maryland School of Medicine, Baltimore, Maryland 21201, USA. asulakve@medicine.umaryland.edu

## Abstract

In May 1998, three large outbreaks of salmonellosis, affecting 91 persons, were identified in the Republic of Georgia. Eighteen Salmonella Typhimurium strains were characterized by arbitrary primed polymerase chain reaction and pulsed-field gel electrophoresis; the results suggested that all cases were part of a single outbreak caused by a distinct clonal strain.


### Salmonellosis in the Republic of Georgia: Using Molecular Typing to Identify the Outbreak-Causing Strain

**Alexander Sulakvelidze,* Merab Kekelidze,† Durmishkhan Turabelidze,* Shota Tsanava,† Lia Tevsadze,† Lamara Devdariani,† Romesh Gautom,‡ Robert Myers,§ J. Glenn Morris, Jr.,* and Paata Imnadze†**

*University of Maryland School of Medicine, Baltimore, Maryland, USA; †National Center for Disease Control, Tbilisi, Republic of Georgia; ‡Public Health Laboratories, Department of Health, Seattle, Washington, USA; and §Maryland Department of Health and Mental Hygiene Laboratories, Baltimore, Maryland, USA

In May 1998, three large outbreaks of salmonellosis, affecting 91 persons, were identified in the Republic of Georgia. Eighteen *Salmonella* Typhimurium strains were characterized by arbitrary primed polymerase chain reaction and pulsed-field gel electrophoresis; the results suggested that all cases were part of a single outbreak caused by a distinct clonal strain.

*Salmonella* species, which cause a variety of clinical manifestations from mild gastroenteritis to septicemia, are one of the leading causes of foodborne illness worldwide (1-2). Approximately 50% of cases of human disease caused by salmonellae are produced by *S.* Enteritidis and *S.* Typhimurium (3). *S.* Typhimurium is of particular concern because of the recent emergence of a highly antibiotic-resistant strain (resistant to ampicillin, chloramphenicol, streptomycin, sulfonamides, and tetracycline) designated as definitive type (DT) 104 (4). In approximately 75% of human *Salmonella* cases, the bacterium is acquired from meat, poultry, or eggs (2,3).

### The Study

In May 1998, three apparently distinct outbreaks of salmonellosis were identified in the Republic of Georgia. Symptoms in all three included acute diarrhea (100% of cases), severe stomach cramps (35%), nausea and vomiting (22%), and fever (97%). The first outbreak affected children attending a birthday party in Kojori, a suburb near Tbilisi, Georgia. Ten of 14 children attending the party came down with acute diarrhea and were hospitalized within 3 days. *S.* Typhimurium was isolated from stool samples of four patients.

The second outbreak, 4 days later, affected guests at a wedding reception in the village of Asureti (40 kilometers east of Tbilisi). Fifty of approximately 100 guests had diarrhea, and 18 were hospitalized. Salmonellosis was confirmed by the isolation of *S.* Typhimurium from seven of the hospitalized patients.

The third outbreak occurred in Tbilisi approximately 14 days after the first outbreak. Thirty-one of 50 guests at a birthday party were hospitalized for acute diarrhea within 3 days. Salmonellosis was culture-confirmed in 14 cases (i.e., *S.* Typhimurium was isolated from 14 of the patients). All persons with diarrhea in all three outbreaks took various doses of antibiotics (ciprofloxacin or ceftriaxone) when they first became ill.

All 59 hospitalized persons were interviewed to determine a possible common source of infection. In addition, 32 persons who had diarrhea but were not hospitalized and 71 healthy guests were interviewed. Eggs were

Address for correspondence: Alexander Sulakvelidze, Division of Hospital Epidemiology, University of Maryland School of Medicine, MSTF Bldg., Room 9-34, 10 S. Pine Street, Baltimore, Maryland 21201, USA; fax: 410-706-4581; e-mail: asulakve@medicine.umaryland.edu.

implicated in the first outbreak; the eggs were used to prepare uncooked icing for a homemade cake. In the second outbreak, eating chicken served during the wedding banquet was associated with illness. In the third outbreak, chicken from a farmers' market in Tbilisi was implicated. No food samples were available for bacteriologic analysis.

Stool samples from the 59 hospitalized patients were examined for *Salmonella*, *Shigella*, and *Yersinia* by standard techniques. *Salmonella* were isolated from stool samples of 25 (42%) of the patients; all 25 isolates were identified as *S.* Typhimurium strains by the API20E test system, and 18 of them were randomly chosen for subsequent analysis. No other pathogen was isolated.

Arbitrary primed polymerase chain reaction (AP-PCR) was performed by using an RAPD kit (Amersham Pharmacia Biotech, Piscataway, NJ). All typing was done with primer #6 of the kit (5'- CCC GTC AGC A - 3'), which gave distinctive, reproducible patterns with three or more major bands. Bacterial DNA for AP-PCR was obtained, and amplification was performed (5). After the amplification cycles, the samples were incubated at 72°C for 5 minutes and analyzed by electrophoresis in 2% agarose gel in TAE buffer.

The rapid pulse-field gel electrophoresis (PFGE) procedure developed for typing *Escherichia coli* O157H7 strains was used for PFGE typing of the outbreak strains (6). The strains were analyzed, in separate experiments, by digesting DNA with *Xba* I, *Avr* II, and *Spe* I restriction enzymes, and *Xba* I-digested *S.* Newport strain amO1144 was used as the reference strain in all experiments (Figure 1).

A distinct pattern was observed in all 18 strains when they were analyzed by AP-PCR (data not shown), which suggests close genetic relatedness. This finding was confirmed by PFGE typing, i.e., *Xba* I-, *Avr* II-; *Spe* I-macrorestriction patterns generated by PFGE showed that all strains tested had an identical PFGE pattern. The pattern was distinct from those obtained for 18 other *S.* Typhimurium strains isolated in the Republic of Georgia at about the same time, which were grouped into five PFGE types distinct from that of the outbreak-causing strain.

The national molecular subtyping network, PulseNet, includes a database of the PFGE patterns of *E. coli* O157 and *Salmonella* group B and D strains isolated in the United States. We screened local databases of two public health laboratories in Washington and Maryland that participate in PulseNet, and we used the Internet to compare the PFGE pattern of the Georgian outbreak strain with *Salmonella* patterns obtained in these two laboratories. At least three strains among >300 *S.* Typhimurium isolates (grouped into approximately 50 PFGE types) in the two databases were almost identical to the Georgian isolate. However, the PFGE patterns of the strains differed slightly when their DNA was digested with *Avr* II before PFGE. The patterns obtained by AP-PCR and PFGE (*Xba* I- and *Spe* I-digests) were almost indistinguishable; however, when DNA of the same strains was analyzed by PFGE after digestion with *Avr* II, differences were detected. For example, the PFGE pattern of strain 00354 (a Seattle isolate) had a large fragment not present in the Georgian outbreak strain and had lost one small fragment (Figure 2, lane 5). In addition, the PFGE pattern of strain 01587 (also

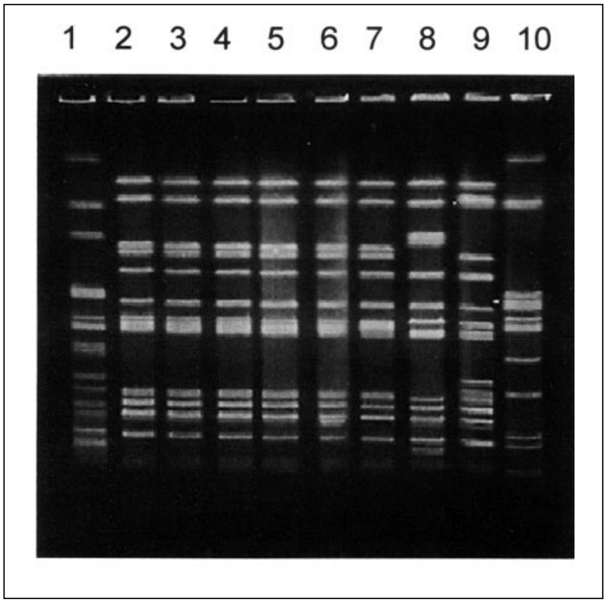

Figure 1. Pulse-field gel electrophoresis patterns of *Xba* I-digested DNA of *Salmonella* Typhimurium strains. Lane 1, *Xba* I-digested *S.* Newport control strain am01144; lanes 2 through 4, *S.* Typhimurium strains isolated during the first, second, and third outbreaks in Georgia, respectively; lane 5, strain 00354 (Washington isolate); lane 6, strain 01587 (Washington isolate); lane 7, 9294-99 (Maryland isolate); lanes 8, 9, and 10, genetically unrelated control *S.* Typhimurium strains isolated in Maryland, Washington, and the Republic of Georgia, respectively.

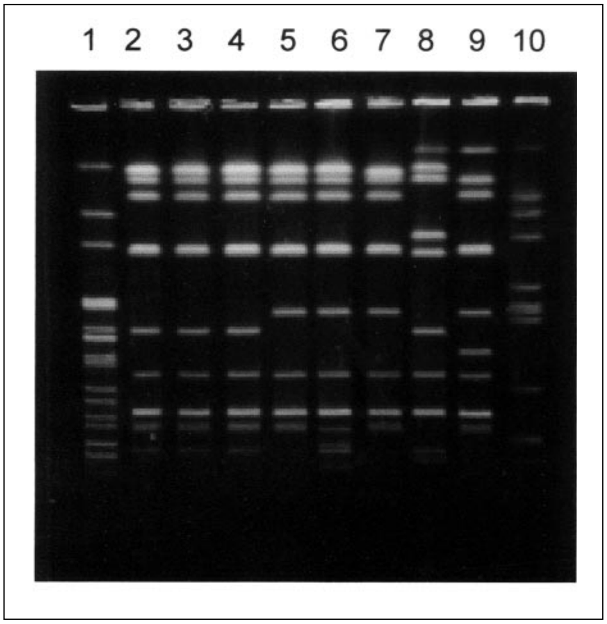

Figure 2. Pulse-field gel electrophoresis patterns of *Avr* II-digested DNA of *Salmonella* Typhimurium strains. Lane 1, *S.* Newport control strain am01144 (*Xba* I-digest); lanes 2 through 4, *S.* Typhimurium strains isolated during the first, second, and third outbreaks in Georgia, respectively; lane 5, strain 00354 (Washington isolate); lane 6, strain 01587 (Washington isolate); lane 7, 9294-99 (Maryland isolate); lanes 8, 9, and 10, genetically unrelated control *S.* Typhimurium strains isolated in Maryland, Washington, and the Republic of Georgia, respectively.

from Seattle) lacked one fragment present in the Georgian outbreak strain and had two other small fragments (Figure 2, lane 6). These differences may result from a single genetic event in the bacterial DNA and are associated with a spontaneous point mutation resulting in either creation or loss of a restriction site. Therefore, the Seattle and Maryland isolates were classified as closely related (7,8) to the Georgian outbreak strain. In Washington, the strain was associated with severe diarrhea like that of the Georgian patients, which suggests that the strain was strongly virulent. (Clinical data for the Maryland isolate were not available.) Detailed evaluation of the pathogenic potential of the outbreak strain (and the closely related U.S. isolates) will require determination of the possible links between unusually severe cases of salmonellosis and isolation of *S.* Typhimurium strains having closely related PFGE patterns, and testing the strain for virulence in laboratory animals.

The outbreak-causing *S.* Typhimurium strain was resistant to ampicillin, chloramphenicol, streptomycin, and tetracycline, but was susceptible to cephalosporins and sulfonamides. Two of the above closely related strains (00354 and 9294-99) had an antibiotic-susceptibility pattern similar to that of the Georgian outbreak strain. The only difference in the pattern for the third closely related strain (01587) was that it was susceptible to chloramphenicol.

### Conclusions

Epidemiologically, the outbreaks had no obvious connection. Patients were hospitalized in two different hospitals in two different cities and were not associated with more than one outbreak. In addition, no common source of infection could be identified. Thus, the three outbreaks were initially thought to be separate. However, the observation that they occurred within a short period (2 weeks between the first and third outbreaks), were caused by the same serotype of *Salmonella*, were associated with severe diarrhea, and were limited geographically to a 80-kilometer radius raised the possibility that the outbreaks may have been related. Therefore, we used molecular typing techniques to characterize the *Salmonella* strains isolated from the patients in each outbreak. Our observations suggest that what appeared to be three distinct outbreaks of salmonellosis were, in reality, parts of one large outbreak caused by a distinct clonal strain of *S.* Typhimurium. The common source of infection and the transmission route for the outbreak-causing *Salmonella* strain are not known.

The clinical diagnosis of salmonellosis was confirmed by isolating *S.* Typhimurium from 25 (42%) of the hospitalized patients. All the patients had treated themselves with ciprofloxacin and ceftriaxone (readily available without prescription in Georgia) before hospitalization, which may have contributed to our inability to isolate *Salmonella* (or other potential pathogens) from most of them. Fifty-nine (65%) of 91 persons diagnosed as having salmonellosis had diarrhea severe enough to require hospitalization. The hospitalization rate of these patients was approximately seven times higher than the usual hospitalization rate (<10%) for *Salmonella* cases in Georgia and approximately three times higher than the average hospitalization rate reported for *Salmonella* patients in the United

States (9). Although some persons with diarrhea may have requested hospitalization after hearing that other ill guests were hospitalized, the clinical reports indicate that the high hospitalization rate reflects a severe manifestation of the disease. Since the AP-PCR and PFGE typing results suggested that a single clone was the causative agent in all cases, the question of its possibly increased virulence arose. It has recently been reported (J.G. Morris, et al., unpub. data) that some *Salmonella* serotypes/ strains cause more severe illness. The recent emergence of *S.* Typhimurium DT104 strains having increased virulence (4), in addition to being multidrug resistant, also highlights the possibility of supervirulent strains emerging worldwide.

*S.* Typhimurium, in contrast to *S.* Enteritidis, which is a highly clonal organism (5), is a fairly diverse serotype (10). Therefore, detection of closely related *S.* Typhimurium strains in geographically distinct loci (the Republic of Georgia, and the East Coast and West Coast of the United States) may signal worldwide spread or emergence of closely related clonal groups of *Salmonella* having increased virulence. This possibility may be confirmed by worldwide (or nationwide) standardization of molecular typing protocols and further strengthening of data-sharing capabilities between laboratories involved in the molecular typing of pathogenic microorganisms. An example demonstrating the potential value of such a database is our finding, during screening of the two participating PulseNet laboratories, that two seemingly unrelated strains (Seattle isolate 00354 and Maryland strain 9294-99) were clonal. As described above, this observation was confirmed by both antibiotic-susceptibility testing and side-by-side molecular typing of the strains in question. An epidemiologic link between the two isolates is being investigated.

### Acknowledgments

The authors thank Arnold Kreger for his helpful discussions and editorial comments and Lia Tsintsadze, Judith Johnson, and Sadaf Qaiyumi for their help in determining the antibiotic susceptibility and resistance of selected *Salmonella* strains used in this study.

The study was funded in part by the International Training and Research in Emerging Infectious Diseases grant from the Fogarty International Center, National Institutes of Health.

Dr. Sulakvelidze is assistant professor of Medicine at the University of Maryland School of Medicine. His research interests include molecular epidemiology and the pathogenesis of diseases caused by bacterial enteric pathogens and toxins. He is also involved in studies evaluating the utility of bacteriophages in the prophylaxis and treatment of diseases caused by various multidrug-resistant bacteria.

### References

1. Goldberg MB, Rubin RH. The spectrum of *Salmonella* infection. Infect Dis Clin N Am 1988;2:571-98.
2. Hook EW. *Salmonella* species (including typhoid fever). In: Mandell GL, Douglas RG, Bennett JE, editors. Principles and practice of infectious diseases. New York: Churchill Livingstone; 1990.
3. Centers for Disease Control and Prevention. *Salmonella* surveillance, annual summary, 1993-1995. Atlanta (GA): The Centers; 1996.
4. Centers for Disease Control and Prevention. Multidrug resistant *Salmonella* serotype typhimurium—United States. MMWR Morb Mortal Wkly Rep 1997;46:308-10.
5. Lin AW, Usera MA, Barrett TJ, Goldsby RA. Application of random amplified polymorphic DNA analysis to differentiate strains of *Salmonella enteritidis*. J Clin Microbiol 1996;34:870-6.
6. Gautom RK. Rapid pulsed-field gel electrophoresis protocol for typing of *Escherichia coli* O157:H7 and other gram-negative organisms in one day. J Clin Microbiol 1997;11:2997-80.
7. Tenover FC, Arbeit RD, Goering RV, Mickelsen PA, Murray BE, Persing DH, et al. Interpreting chromosomal DNA restriction patterns produced by pulsed field gel electrophoresis: criteria for bacterial strain typing. J Clin Microbiol 1995;33:2233-9.
8. Olive MD, Bean P. Principles and applications of methods for DNA-based typing of microbial organisms. J Clin Microbiol 1999;6:1661-9.
9. Mead PS, Slutsker L, Dietz V, McCaig LF, Bresee JS, Shapiro C, et al. Food-related illness and death in the United States. Emerg Infect Dis 1999;5:607-25.
10. Nastasi A, Mammina C, Villafrate MR. Epidemiology of *Salmonella typhimurium*: ribosomal DNA analysis of strains from human and animal sources. Epidemiol Infect 1993;3:553-65.
